# Development of new classification criteria for macrophage activation syndrome complicating systemic juvenile idiopathic arthritis

**DOI:** 10.1186/1546-0096-12-S1-O1

**Published:** 2014-09-17

**Authors:** Francesca Minoia, Sergio Davì, Francesca Bovis, Angela Pistorio, Maurizio Aricò, Tadej Avcin, Ed Behrens, Fabrizio De Benedetti, Lisa Filipovich, Alexei Grom, Jan-Inge Henter, AnnaCarin Horne, Norman Ilowite, Michael Jordan, Raju Khubchandani, Toshiyuki Kitoh, Kai Lehmberg, Dan Lovell, Paivi Miettunen, Kim Nichols, Jana Pachlopnik, Seza Ozen, Athimalaipet V Ramanan, Ricardo Russo, Rayfel Schneider, Gary Sterba, Carol Wallace, Carine Wouters, Nico Wulffraat, Yosef Uziel, Nicola Ruperto, Alberto Martini, Randy Q Cron, Angelo Ravelli

**Affiliations:** 1 Istituto G. Gaslini, Genova, Italy; 2 Investigator Consortium for MAS Classification Criteria (ICMCC) , Firenze, Italy; 3 ICMCC, Ljubljana, Slovenia; 4 ICMCC, Philadelphia, USA; 5 ICMCC, Roma, Italy; 6 ICMCC, Cincinnati, USA; 7 ICMCC, Stockholm, Sweden; 8 ICMCC, New York, USA; 9 ICMCC, Mumbai, India; 10 ICMCC, Nagakute, Japan; 11 ICMCC, Hamburg, Germany; 12 ICMCC, Calgary, Canada; 13 ICMCC, Zurich, Switzerland; 14 ICMCC, Ankara, Turkey; 15 ICMCC, Bristol, UK; 16 ICMCC, Buenos Aires , Argentina; 17 ICMCC, Toronto, Canada; 18 ICMCC, Caracas, Venezuela, Bolivarian Republic Of; 19 ICMCC, Seattle, USA; 20 ICMCC, Leuven, Belgium; 21 ICMCC, Utrecht, Netherlands; 22 ICMCC, Kfar Saba, Israel; 23 ICMCC, Birmingham, USA

## Introduction

Macrophage activation syndrome (MAS) is a potentially fatal complication of systemic juvenile idiopathic arthritis (sJIA), whose prompt recognition and treatment are critical. However, early diagnosis of MAS is often challenging and none of the current diagnostic criteria is satisfactory. An international project aimed to develop a new set of classification criteria for MAS was recently started.

## Objectives

To present the results of the consensus conference that led to the development of the new classification criteria for MAS complicating sJIA.

## Methods

28 pediatric specialists (20 rheumatologists and 8 hemato-oncologists) with expertise in MAS reviewed 428 profiles of patients with sJIA-associated MAS or with a confusable condition (active sJIA or systemic infection). The expert panel classified each patient as having or not having MAS based on clinical and laboratory features at disease onset. Using the experts’ consensus as “gold standard”, a statistician tested 982 candidate definitions, derived from both literature and statistical analyses. Definitions with a kappa level of agreement ≥ 0.85 were included in the expert voting process during the consensus conference. In a secondary analysis, experts were asked to declare whether the change in laboratory parameters over time was consistent or not with MAS and to rank laboratory tests in order of the importance of their change in the diagnosis of MAS.

## Results

After 5 rounds, experts achieved consensus on approximately 90% (391/428) of the profiles submitted. Statistical analyses led to select 45 definitions with kappa ≥ 0.85. During the consensus conference, 7 voting sessions were made. Finally consensus (82%) was reached on the following definition: “A febrile patient with known or suspected sJIA is classified as having MAS if the patient has: ferritin >684 ng/mL and at least 2 of the following 4 laboratory abnormalities: platelets ≤ 181 x 10^9^ /L, aspartate aminotransferase (AST) > 48 U/L, triglycerides > 156 mg/dL, and fibrinogen ≤ 360 mg/dL”. In the evaluation of change, falling platelet count, hyperferritinemia and increased AST received the highest scores.

## Conclusion

A new set of classification criteria for MAS complicating sJIA was agreed upon in a multinational consensus conference, which gathered the leading experts in the field. The new criteria deserve validation in a new cohort of patients with MAS seen prospectively.

## Disclosure of interest

None declared.

